# Impact of *Lepeophtheirus salmonis* infestations on migrating Atlantic salmon, Salmo salar L., smolts at eight locations in Ireland with an analysis of lice-induced marine mortality

**DOI:** 10.1111/jfd.12054

**Published:** 2013-01-09

**Authors:** D Jackson, D Cotter, J Newell, S McEvoy, P O'Donohoe, F Kane, T McDermott, S Kelly, A Drumm

**Affiliations:** 1 Marine Environment and Food Safety Services, Marine InstituteGalway, Ireland; 2 School of Mathematics, Statistics and Applied Mathematics, National University of IrelandGalway, Ireland

**Keywords:** Atlantic salmon, *Lepeophtheirus salmonis*, mortality, sea lice

## Abstract

Sea lice infestation as a source of marine mortality of outwardly migrating Atlantic salmon smolts has been investigated by treating groups of ranched salmon, prior to release, with a prophylactic sea lice treatment conferring protection from sea lice infestation. A number of studies have been carried out in Ireland using both established ranched populations and groups of hatchery reared fish imprinted for 5–8 weeks in the sites of experimental releases. In this study, data on 352 142 migrating salmon from twenty-eight releases, at eight locations along Ireland's South and West coasts covering a 9-year period (2001 to 2009) are reviewed. Both published and new data are presented including a previously unpublished time series. The results of a meta-analysis of the combined data suggest that while sea lice-induced mortality on outwardly migrating smolts can be significant, it is a minor and irregular component of marine mortality in the stocks studied and is unlikely to be a significant factor influencing conservation status of salmon stocks.

## Introduction

Across the North Atlantic region estimates of prefishery abundance of Atlantic salmon, *Salmo salar* L., developed by ICES indicate marked declines. The decline from the early 1970s to the mid-1990s in one-sea-winter stocks has been estimated at about 46% and in multi-sea-winter stocks at 65% (Hutchinson & Mills 2000). Declines in marine survival of Atlantic salmon have been recorded in Ireland (Salmon Management Task Force Report (Anon. 1996); Ó Maoiléidigh *et al*. 2004; Jackson *et al*. 2011a) and elsewhere in European and North American stocks (Baum 1997; Hutchinson & Mills 2000). The reasons for the trend towards reduced sea survival remains unclear and global warming effects (Friedland *et al*. 2000; Friedland, Chaput & MacLean 2005), changes in locations or availability of prey species associated with the North Atlantic oscillation (Reid & Planque 2000), loss of post-smolts as by-catch in pelagic fisheries, increased fishing pressure, predation, habitat changes, sea lice infestation (Finstad *et al*. 2007) and sea lice-induced mortality (Gargan *et al*. 2012) have been suggested.

The Marine Institute has undertaken a long-term study since 2001 to investigate if sea lice infestations were a significant factor in early marine mortality of Irish salmon smolts and to measure the inter-annual variation in the impacts of early sea lice infestations on sea survival. A number of aspects of this work using established ranched strains have been published (Jackson *et al*. 2011a,b). The goal of the present article is to analyse the data from a large number of fish (in excess of 350 000) involving both multiple river systems and multiple releases covering almost a decade to take account of the inherent variability in salmon survival while assessing the extent of sea lice-induced mortality in Irish Atlantic salmon stocks.

## Materials and methods

### Experimental design

**Figure 1:**
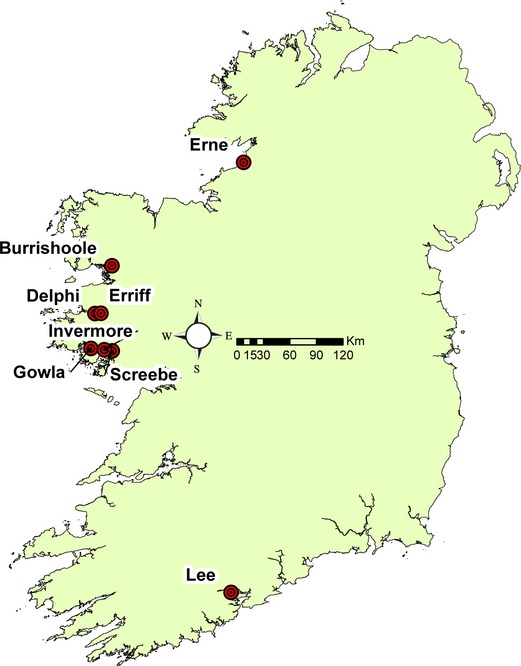
Locations of release points.

By treating experimental batches of tagged fish, prior to release, with a prophylactic dose of SLICE®, a commercial sea lice therapeutant, the fish can be protected from infestation with the salmon louse, *Lepeophtheirus salmonis* Kroyer, for up to 9 weeks (Copley *et al*. 2007; Jackson *et al*. 2011b). The active ingredient in SLICE® is emamectin benzoate. It is an animal medicine licensed for use in Ireland as a treatment for sea lice infestation in salmon. As salmon smolts are known to migrate quickly out of the bays and into the open sea, treated smolts will have moved well offshore before the protective effects of the SLICE® treatment have worn off. Studies at Burrishoole have shown that salmon smolts have moved into coastal waters within 48 h (Moore *et al*. 2008) and post-smolt recapture data (Shelton *et al*. 1997; Dadswell *et al*. 2010) have shown that smolts from the study area have travelled a distance of over 700 kilometres in 7 weeks and are in an area north of Scotland and west of Norway. By comparing return rates of treated fish with untreated control fish, it is possible to differentiate any additional mortality associated with sea lice infestation in the first 6–8 weeks post-migration (Jackson *et al*. 2011a). This methodology has been employed on a series of releases of ranched stocks from the Burrishoole river, the Bundorragha river (Delphi) and at a number of other locations (Fig. 1) on Ireland's south and west coast (Jackson *et al*. 2011a,b). In addition, data published by Gargan *et al*. (2012) using hatchery stocks transplanted into salmon and sea trout rivers and imprinted there for 5–8 weeks has been included in the analysis together with previously unpublished data from both Burrishoole and Bundorragha (Delphi).

### Tagging, tag recovery and data analysis

Experimental batches of fish were all tagged with coded wire tags. Presmolts were microtagged according to the methods of Browne (1982), whereby a 1 mm long magnetized tag, etched with a specific batch code was injected into the nose cartilage of the juvenile fish. The code identifies the origin and release circumstances of any fish subsequently recaptured. All fish were anaesthetized when tagged, and the adipose fin was removed to facilitate the identification of these fish in the recovery programme. A quality control check was made on the tagged fish to ensure that the tag had been correctly magnetized. Tagging mortality and tag loss were also estimated, and subsequent analyses were based on the number of fish migrating rather than the number of fish tagged. Information on capture location and return data of the experimental groups was gathered as part of an ongoing Irish national coded wire tag recovery programme (Browne *et al*. 1994; Ó Maoiléidigh *et al*. 2004). Prior to 2007 catches from coastal commercial fisheries (drift nets, draft nets, etc.) were monitored at 15 major salmon landing ports in Ireland. These fisheries operate between May and July inclusive, and catches were scanned consistently during this period. Over 50% of the catch landed in Ireland was sampled for tags each year. The number of tagged salmon taken in these fisheries (raised data) was estimated by multiplying the actual number of tagged salmon in each area by the ratio of the total declared salmon landings in these areas to the sample size examined. An adjustment for non-catch fishing mortality due to losses from nets and non-reporting of catches was also applied as part of this process. This methodology, as used in the compilation of returns for ICES and NASCO, ensures the avoidance of sampling bias and the comparability of data with other national and international estimates of marine survival.

### Analyses

Two-way contingency tables were used to calculate expected returns for comparison against observed returns for each pair of treatment and control batches using the chi-squared test. The resultant *P* values were corrected using the Bonferroni procedure for multiple tests. Regression lines with 95% confidence intervals were fitted to the data set for the treated and control groups as a first step in evaluating the trends in the data. A scatterplot of percentages with a Lowess smoother was found to give a more appropriate visual representation of the data.

### Statistical analyses

The primary analysis was carried out using the generalized logistic model, and then a secondary analysis was carried out by treating the percentages as continuous (weighted) response variables.

Comparing the percentage returning without adjusting for the fact that the percentages represent considerably different denominators limits the discriminatory power of the analysis. To overcome this one needs to allow a comparison of the proportion of fish returning (i.e. a binomial response variable) between the treated and control groups to be made, while adjusting for release year and river location and for the differing number of fish migrating for each treatment/release year/location combination. A logistic regression model was fitted to model the probability of returning as a function of treatment group and release time (and their interaction) while adjusting for the association between fish released from the same location and for the differing numbers migrating from each location and year. The best model identified was one containing an interaction between release year and treatment to adjust for the fact that the positive effect of the treatment differed across release years. A generalized mixed model was fitted to the data by the Laplace approximation, and model diagnostics were carried out by examining plots of residuals and fitted values for goodness of fit.

A linear model (i.e. an analysis of variance) was fitted where the response variable was the percentage returns (weighted by migration) with treatment, location and release date as factors. Initially, a model containing all two- and three-way interactions between the factors was fitted, and then non-significant terms were removed based on backwards elimination.

## Results

The release locations are shown in Fig. 1, and the release groups, dates of release with numbers and return rates are given in Table 1 together with *P* values for significance. After correction using the Bonferroni adjustment, 11 of the 28 release groups or approximately 40% showed a significant difference in return rate between treated and control groups.

**Table 1:** Summary data on release groups of salmon including chi-squared value and *P* value with Bonferroni correction

			Control			Slice			Chi-squared Test
					
Group Name	Release date	Reference	Estimated Migration number	Raised Returns	Control	Estimated Migration number	Raised Returns	Slice	*P*-value
Delphi 01 BUR	02/05/2001	Jackson *et al*. 2011a	6385	984.8	15.55	6392	1216.6	19.11	<0.001*
Delphi 01 DEL	02/05/2001	Jackson *et al*. 2011a	6395	892.2	14.11	6368	836.1	13.24	0.176
Burr 01	03/05/2001	Jackson *et al*. 2011b	10039	996.6	9.88	5496	565.1	10.28	0.487
Burr 02	01/05/2002	Jackson *et al*. 2011b	5989	542.3	9.10	5960	543.7	9.12	0.89
Gowla 03	28/04/2003	Gargan *et al*. 2012	4822	20.4	0.42	4955	225.6	4.55	<0.001*
Invermore 03	29/04/2003	Gargan *et al*. 2012	4594	37.7	0.82	4589	88.6	1.93	<0.001*
Burr 03	01/05/2003	Jackson *et al*. 2011b	4587	373.8	8.15	4745	471.1	9.93	0.003
Gowla 04	07/04/2004	Gargan *et al*. 2012	4699	91.0	1.94	4655	164.6	3.54	<0.001*
Invermore 04	08/04/2004	Gargan *et al*. 2012	4671	96.2	2.06	4653	105.4	2.27	0.484
Erriff 04	12/04/2004	Gargan *et al*. 2012	4229	107.9	2.55	4325	101.8	2.35	0.551
Burr 04	29/04/2004	Jackson *et al*. 2011b	4369	398.2	9.11	4437	403.3	9.07	0.974
Erriff 05	04/04/2005	Gargan *et al*. 2012	4689	8.4	0.18	4659	171.8	3.69	<0.001*
Gowla 05	07/04/2005	Gargan *et al*. 2012	4735	317.8	6.71	4583	306.3	6.68	0.95
Invermore 05	08/04/2005	Gargan *et al*. 2012	4750	111.2	2.34	4716	195.8	4.15	<0.001*
Delphi 05	26/04/2005		8893	831.1	9.35	8471	1038.4	12.26	<0.001*
Burr 05	28/04/2005	Jackson *et al*. 2011b	3867	183.2	4.71	3793	253.0	6.67	<0.001*
Lee 06	04/04/2006	Jackson *et al*. 2011a	5131	10.0	0.19	5207	10.0	0.19	0.974
Burr 06 Apr	26/04/2006	Jackson *et al*. 2011b	4779	211.0	4.44	4809	326.0	6.82	<0.001*
Screebe 06	28/04/2006	Jackson *et al*. 2011a	9618	121.0	1.26	10990	157.0	1.43	0.29
Delphi 06	29/04/2006		8788	172.4	1.96	10560	477.9	4.53	<0.001*
Burr 06 May	04/05/2006	Jackson *et al*. 2011b	8000	334.0	4.21	3907	180.0	4.61	0.276
Erne 06	04/05/2006	Jackson *et al*. 2011a	10357	68.0	0.66	5752	70.0	1.22	<0.001*
Burr07	24/04/2007	Jackson *et al*. 2011b	6784	440	6.40	6746	491	7.29	0.069
Delphi 07	26/04/2007		9719	567.4	5.84	9451	550.8	5.83	0.986
Delphi 08 DEL	28/04/2008		10811	183.0	1.69	16346	293.0	1.79	0.54
Burr 08 Apr	29/04/2008	Jackson *et al*. 2011b	6832	76.0	1.11	6719	97	1.44	0.086
Burr08 May	06/05/2008	Jackson *et al*. 2011b	3392	54.0	1.59	3413	72	2.11	0.113
Burr 09	28/04/2009		6640	300.0	4.47	6881	267	3.88	0.064

* Comparisons that were still significant after a Bonferroni correction.

Of the 352 142 migrating salmon, 18 208 were recovered representing a sample proportion of 5.17% (95% confidence interval 5.1%, 5.2%). The small margin of error in the confidence interval is a consequence of the large sample size. This result suggests that, in the population of salmon represented by the sample provided, between 5.1% and 5.2% of salmon released are likely to return. The average marine mortality over the period of the study is therefore >94%, between 94.8% and 94.9%.

There was a higher proportion of salmon returning in the treated group (5.6%) compared with the controls (4.8%). This represents a difference of approximately 0.8% between the groups favouring those having received SLICE® (i.e. nearly 1% higher returned in the SLICE® group). An interval estimate for the difference in population proportions returning is calculated as 0.6–0.9% (Table 2). As this interval is strictly positive (i.e. does not contain zero), there is evidence that the treatment is having a positive effect in terms of a higher proportion of salmon returning albeit the improvement over the control group or absolute difference in risk is of the magnitude of approximately 1%.

**Table 2:** Test and CI for two proportions

Sample	Returns	Migrated	Sample proportions
Slice	9680	173578	0.055767
Control	8528	178564	0.047759
Difference = prop (Slice)−prop (Control)			
Estimate for difference: 0.00800865			
95% CI for difference: (0.00654450, 0.00947280)			
Test for difference = 0 (vs. not = 0): Z = 10.72; *P*-Value = 0.000			
Fisher's exact test: *P*-Value = 0.000			

**Figure 2:**
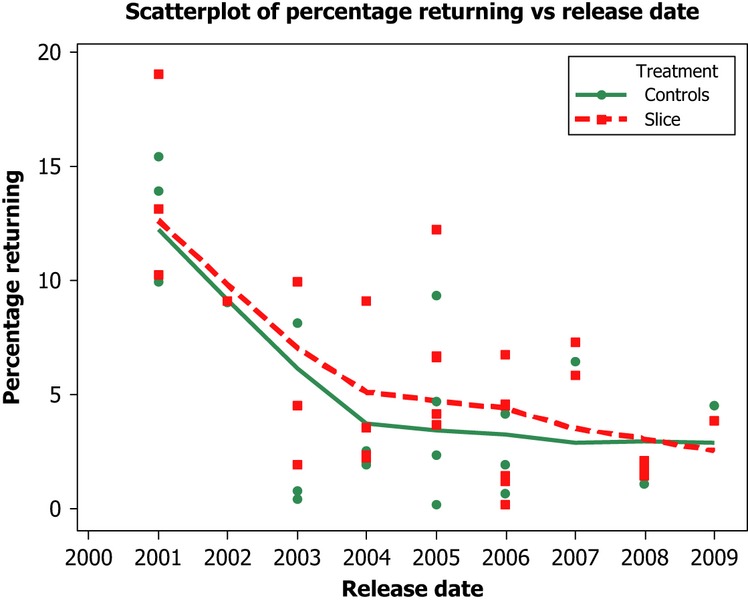
Percentage of salmon returning for each treatment group with Lowess smoother.

**Figure 3:**
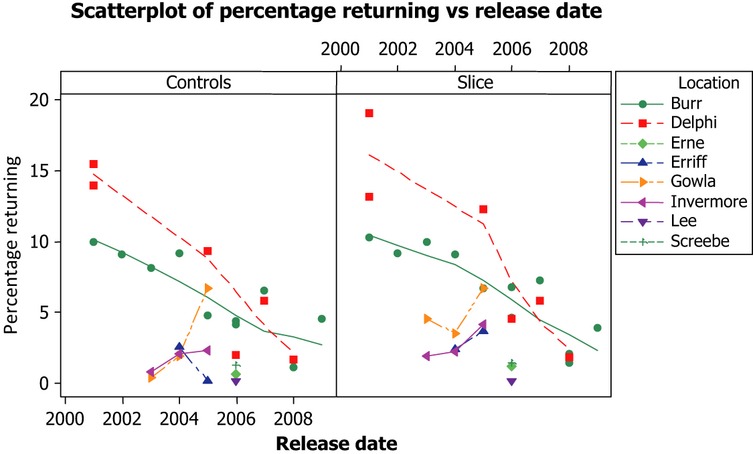
Percentage of salmon returning by location and date with Lowess smoother.

There is considerable variability evident in the proportions returning between the different locations as evident in the numerical summaries in Table 1. A plot of the percentage returning within each Treatment Group by Release Year with a Lowess smoother superimposed is given in Fig. 2. There is a reduction in the percentage returning by year with a large reduction evident from 2001 to 2004. There is a suggestion from the smoother that the proportion returning is higher for the SLICE® group across time but that the magnitude of the difference in proportions between the groups differs across time (i.e. there appears to be a Release Date by Treatment Group interaction). To visually assess the additional effect, if any, of the Location on the proportions returning, a plot of the percentage returning by Release Year and Location panelled by Treatment Group with a Lowess smoother superimposed, is given in Fig. 3. There is evidence that the percentage returning by Year differs between locations. The highest returns were evident in the Bundorragha (Delphi) with the lowest evident in the Erriff. The Bundorragha (Delphi) and Burrishoole appear to have a similar pattern across time and between treatment groups, while the relationship across time and between groups differ considerably for the remaining locations.

The output from the generalized logistic model identified a significant treatment effect (*P* < 0.001). There is evidence of an overall treatment effect favouring the treated group as the estimate is positive (0.13072). The odds of a fish returning are 1.14:1 (95% confidence interval 1.07, 1.21) in favour of the SLICE® group. The estimated probability of a treated fish returning (averaging over all years and rivers) can be calculated from the model as 0.097 compared with an estimated probability of a control fish returning (averaging over all years and rivers) of 0.086, an absolute difference of 0.011. This is approximately 1% or 10 fish in a thousand. Running the model for the Bundorragha (Delphi) and Burrishoole time series of data combined also gave a significant treatment effect (*P* < 0.001). Running the analysis for the Bundorragha (Delphi) alone gave a significant (*P* = 0.001) treatment effect but not for the Burrishoole time series (*P* = 0.49). As sample size is reduced the smaller numbers may be driving the lack of significance.

**Figure 4:**
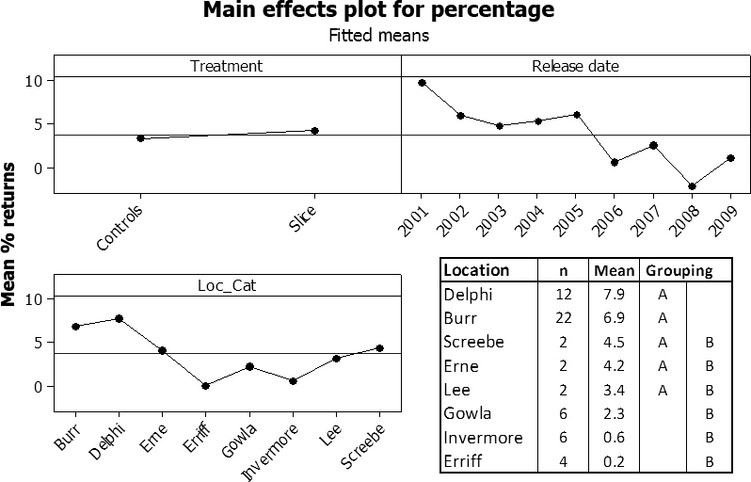
ANOVA: plot of adjusted mean percentages, all data.

**Figure 5:**
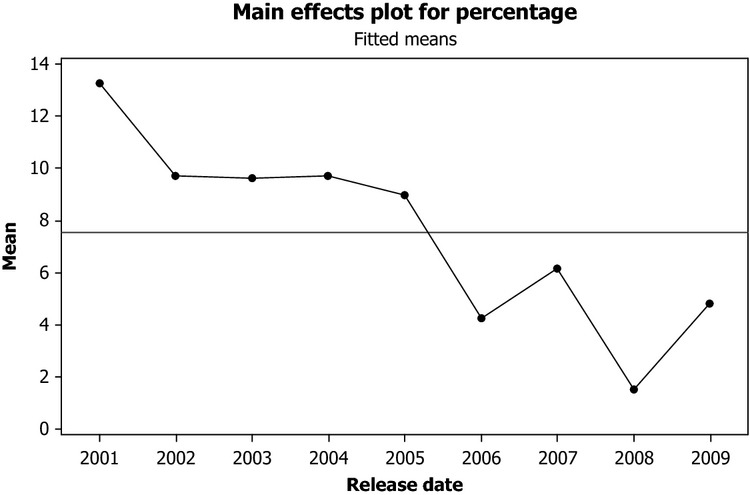
ANOVA plot of adjusted mean percentages, Burrishoole and Bundorragha (Delphi) time series.

The outputs of the linear model (ANOVA) containing main effects only are given in Table 3. There was evidence of significant Treatment (*P* = 0.034), Location (*P* < 0.001) and Release Date (*P* < 0.001) effects. Two observations were identified as unusual, namely the controls in Burrishoole in 2001 where the observed percentage return (9.93%) was considered low for such a control group and for the treated fish originating from Bundorragha (Delphi) in 2001 where the observed percentage return of 19.03% was considered high relative to other treated fish. A plot of the adjusted mean percentages (i.e. those summarized by the model) is given in Fig. 4. As the major effect identified was release date, the ANOVA was run for the two locations with a significant time series, Burrishoole and Bundorragha (Delphi). There was evidence of a significant Release Date (*P* < 0.001) effect but neither Location Effect (*P* = 0.116) nor Treatment Group (*P* = 0.156) was significant (Fig. 5).

**Table 3:** Analysis of variance for percentage, using adjusted SS for tests

Source	df	Seq SS	Adj SS	Adj MS	F	*P*
Location	7	1968060	1302641	186092	10.19	0
Release date	8	3743239	3790018	473752	25.95	0
Treatment	1	88165	88165	88165	4.83	0.034
Error	39	711907	711907	18254		
Total	55	6511371				
*S* = 135.107; R-Sq = 89.07%; R-Sq(adj) = 84.58%						

Obs	Percentage	Fit	SE Fit	Residual	St Resid
1	9.9268	12.329	0.7902	−2.4022	−2.2 R
24	19.033	14.2887	0.7461	4.7444	3.13 R

The plot of the Location Effect (Fig. 4) suggests that the highest returns were in the Bundorragha (Delphi) (adjusted mean percentage was 7.9%) with Erriff having the lowest (adjusted mean percentage was 0.2%). The table of the adjusted means (i.e. adjusting for migration, treatment and release date) identifies locations that have significantly different mean percentages as those that do not share a letter in common. Bundorragha (Delphi) and Burrishoole are comparable while both are significantly different (i.e. higher) than Gowla, Invermore and Erriff. There is no evidence of a difference between Screebe, Erne, Lee, Gowla, Invermore and Erriff.

## Discussion

The data set available for analysis in this study is both large in terms of numbers of fish and comprehensive in terms of temporal and geographic coverage. The large numbers give the resulting analysis great statistical power, with the ability to detect very small differences. In designing the experimental framework (Jackson *et al*. 2011b), cognisance was taken of the synergistic effect of transferring smolts between rivers of different chemical composition (Saunders *et al*. 1983) with short-term exposure to acid waters on survival and straying. Only line bred ranched stocks reared from egg through to smolts and released within a system were used throughout the time series studies in the Delphi and Burrishoole and in the studies presented in Jackson *et al*. (2011b) to ensure consistency in rates of return. In contrast, Gargan *et al*. 2012 transferred ranched presmolts from an alkaline river body (Lough Corrib) to distant acidic rivers. This may account for the markedly lower survival in these groups (Fig. 4), which in certain cases (e.g. Invermore and Erriff) was an order of magnitude lower than the means for the other rivers. Recent research suggests that the effects of acid water (Staurnes *et al*. 1996) and the interactive effects of acidification and salmon lice infestation on post-smolt survival (Finstad *et al*. 2007) result in reduced survival through increased predation and straying. This may limit the value of data based on stocks relocated into acid waters before release.

The temporal and geographic coverage of the data allow for a certain confidence in the results being representative. This confidence is increased by the fact that the declines in survival recorded in this study (Fig. 5) are mirrored in the reported national marine survival trends published in the report of the Standing Scientific Committee of the National Salmon Commission (Anon 2009). Both the analysis of all data (Fig. 4) and the analysis of the time series from the Burrishoole and Bundorragha (Delphi) catchments identify 2008 as the year with the lowest marine survival in the study period and a slight recovery in marine survival in 2009. This concurs with the national trends in marine survival.

The results show a small but significant impact of sea lice infestation on marine survival of outwardly migrating salmon. This represents an absolute difference in returns of approximately 1% in favour of treated fish. This difference was significant in just under 40% of releases using the Chi-squared test with Bonferroni adjustment (Table 1). It was sensitive to sample size, being significant in the analysis of the total data set, but not in the Burrishoole time series alone, which makes up approximately 35–40% (depending on whether you measure this in terms of number of releases or estimated migration number) of the data set (Table 1). The observed level of marine mortality attributable to sea lice infestation is very small, both in absolute terms (approximately 1%) and as a proportion of the overall marine mortality which in this study had a mean value >90% at all locations. At these levels, it is unlikely to influence the conservation status of stocks and is not a significant driver of marine mortality. Recent studies have been carried out in Norway with broadly similar results (Skilbrei & Wennevik 2006).

The results also show a major fall in marine survival over the study period. This is significant in both the combined data (Fig. 4) and in each of the time series of data from Burrishoole and Bundorragha (Delphi). This fall in marine survival is mirrored in both the treated and control groups and is the main source of variance in the data (*F* = 25.95, Table 3.). When applied to the combined time series for two catchments (Fig. 5), it was the only significant source of variance. The lack of a pattern displayed for the remaining locations may be due to a combination of small sample sizes, increased levels of straying due to use of imprinted non-native stocks and a lower recovery rate for freshwater returns due to incomplete or absent upstream trapping facilities. Previous studies (Jackson *et al*. 2011a,b) have shown that there was no difference between the means of treated and untreated groups at one location (Burrishoole) and that a common regression of both treated and untreated groups was highly significant. That study concluded that infestation of outwardly migrating salmon with *L. salmonis* was not implicated in the observed significant decline in survival rate. The analyses carried out here on a much larger data set with two significant time series would support this conclusion. The declines in both treated and control groups follow similar trends (Fig. 2) and when separated out (Fig. 3), it can be seen that both the Bundorragha (Delphi) and Burrishoole data follow similar trends over time and across treated and untreated groups.

The marine survival data in this study reflect the reported national data on marine survival rates for wild salmon in Ireland. There is a strong and significant trend in increasing marine mortality up to 2008. There is no evidence to suggest that this trend is influenced by sea lice infestation levels of outwardly migrating smolts as treated and control fish are equally affected. Sea lice-induced mortality is significant in just under 40% of the releases in the study. The level of sea lice-induced mortality is small as a proportion of the overall marine mortality rate, which is in the region of 90%, and in absolute terms represents 1% (10 fish in a thousand).
